# Carbon-fibre plates for traumatic and (impending) pathological fracture fixation: Where do we stand? A systematic review

**DOI:** 10.1186/s10195-023-00724-4

**Published:** 2023-08-11

**Authors:** Zeger Rijs, Amber Weekhout, Stef Daniel, Jan W. Schoones, Olivier Q. Groot, Santiago A. Lozano-Calderon, Michiel A. J. van de Sande

**Affiliations:** 1 Department of Orthopaedic Surgery, Leiden University Medical Center, Leiden, The Netherlands; 2 Directorate of Research Policy, Leiden University Medical Center, Leiden, The Netherlands; 3 Department of Orthopaedics, Massachusetts General Hospital—Harvard Medical School, Boston, USA

**Keywords:** Fracture fixation, CFR PEEK, Carbon-fibre plates, Complications

## Abstract

**Background:**

Carbon-fibre (CF) plates are increasingly used for fracture fixation. This systematic review evaluated complications associated with CF plate fixation. It also compared outcomes of patients treated with CF plates versus metal plates, aiming to determine if CF plates offered comparable results. The study hypothesized that CF plates display similar complication rates and clinical outcomes as metal plates for fracture fixation.

**Methods:**

The study adhered to the Preferred Reporting Items for Systematic Reviews and Meta-Analyses guidelines. The following databases were searched from database inception until June 2023: PubMed, MEDLINE, Embase, Web of Science, Cochrane Library, Emcare, Academic Search Premier and Google Scholar. Studies reporting on clinical and radiological outcomes of patients treated with CF plates for traumatic fractures and (impending) pathological fractures were included. Study quality was assessed, and complications were documented as number and percentage per anatomic region.

**Results:**

A total of 27 studies of moderate to very low quality of evidence were included. Of these, 22 studies (800 patients, median follow-up 12 months) focused on traumatic fractures, and 5 studies (102 patients, median follow-up 12 months) on (impending) pathological fractures. A total of 11 studies (497 patients, median follow-up 16 months) compared CF plates with metal plates. Regarding traumatic fractures, the following complications were mostly reported: soft tissue complications (52 out of 391; 13%) for the humerus, structural complications (6 out of 291; 2%) for the distal radius, nonunion and structural complication (1 out of 34; 3%) for the femur, and infection (4 out of 104; 4%) for the ankle. For (impending) pathological fractures, the most frequently reported complications were infections (2 out of 14; 14%) for the humerus and structural complication (6 out of 86; 7%) for the femur/tibia. Comparative studies reported mixed results, although the majority (7 out of 11; 64%) reported no significant differences in clinical or radiological outcomes between patients treated with CF or metal plates.

**Conclusion:**

This systematic review did not reveal a concerning number of complications related to CF plate fixation. Comparative studies showed no significant differences between CF plates and metal plates for traumatic fracture fixation. Therefore, CF plates appear to be a viable alternative to metal plates. However, high-quality randomized controlled trials (RCTs) with long-term follow-up are strongly recommended to provide additional evidence supporting the use of CF plates.

*Level of evidence*: III, systematic review.

## Introduction

Carbon-fibre (CF) plates, reinforced with polyetheretherketone, have gained increasing interest due to potential advantages compared with metal plates. For instance, CF plates offer radiolucency, which enables better radiographic visualization of postoperative fracture reduction, bone healing and surveillance of tumour recurrence for oncological patients [1–4]. Furthermore, the absence of metallic artefacts allows for precise radiotherapy planning and accurate delivery after placement of CF implants [5–7]. Another advantage specific to CF plates may be reduced stress shielding, as their modulus of elasticity closely matches that of cortical bone; 13 gigapascal (GPa) for CF versus 12 GPa for cortical bone [8]. Additionally, in vitro studies on CF plates have demonstrated superior fatigue strength compared with current metal plates; this may potentially enhance bone healing and reduce the risk of complications [8, 9]. Finally, cold welding does not occur in CF plate constructs, which would facilitate easy implant removal [10].

Despite the increasing use of CF plates for fixating traumatic and (impending) pathological fractures, reported experience in the literature remains limited. Previous systematic reviews have primarily focused on comparative studies or specifically examined traumatic distal radius fracture fixation with CF plates [11–13]. In these studies, CF plates were considered as a valid alternative due to comparable results to metal plates [11–13]. However, cohort studies and case reports have identified several disadvantages associated with CF plates that were not mentioned in the aforementioned systematic reviews. Drawbacks include the inability to deform the plate, plate breakage without clear trauma and brittleness when plate breakage occurs [14–17]. Conducting a systematic review that includes all relevant existing evidence would provide a comprehensive overview and is crucial for assessing the safety and effectiveness of CF plates. Therefore, the aim of this systematic review was to evaluate complications associated with CF plate fixation for traumatic and (impending) fracture fixation. It also compared outcomes of patients treated with CF plates versus metal plates, aiming to determine if CF plates offered comparable results. Based on the aforementioned systematic reviews, this study hypothesized that CF plates display similar complication rates and clinical outcomes as metal plates for (pathological) fracture fixation.

## Methods

### Search strategy and study selection

This systematic review was conducted in accordance with the Preferred Reporting Items for Systematic Reviews and Meta-Analyses (PRISMA) guidelines and followed a pre-registered PROSPERO protocol (CRD42021254603) [18]. A medical librarian assisted in developing the search strategy, which was based on the following population, intervention, comparison and outcome (PICO) algorithm: P = patients with traumatic or (impending) pathological fractures, I = CF plate fixation, C = no specific controls or patients treated with metal plates and O = radiological and/or clinical outcomes (including complications). Ultimately, the search was divided into two parts: (1) CF plates used for traumatic fractures and (2) CF plates used for (impending) pathological fractures. The search contained keywords related to “carbon-fiber” and “fracture” for traumatic fractures, and “carbon-fiber” and “bone tumor” for (impending) pathological fractures (Appendix 1). The following databases were reviewed from database inception up to June 2023: PubMed, MEDLINE, Embase, Web of Science, Cochrane Library, Emcare, Academic Search Premier and Google Scholar.

### Eligibility criteria

Eligible study designs included randomized controlled trials (RCTs), cohort studies (with prospective and retrospective designs), case–control studies, cross-sectional studies and case reports. Studies were included if they involved patients with traumatic or (impending) pathological fractures fixated with CF plates. Excluded were meeting abstracts, reviews, editorials, commentaries, surveys, animal-only, in vitro, cadaver or biomechanical studies. No filters or other constraints were used in the database search.

### Study selection

**Fig. 1:**
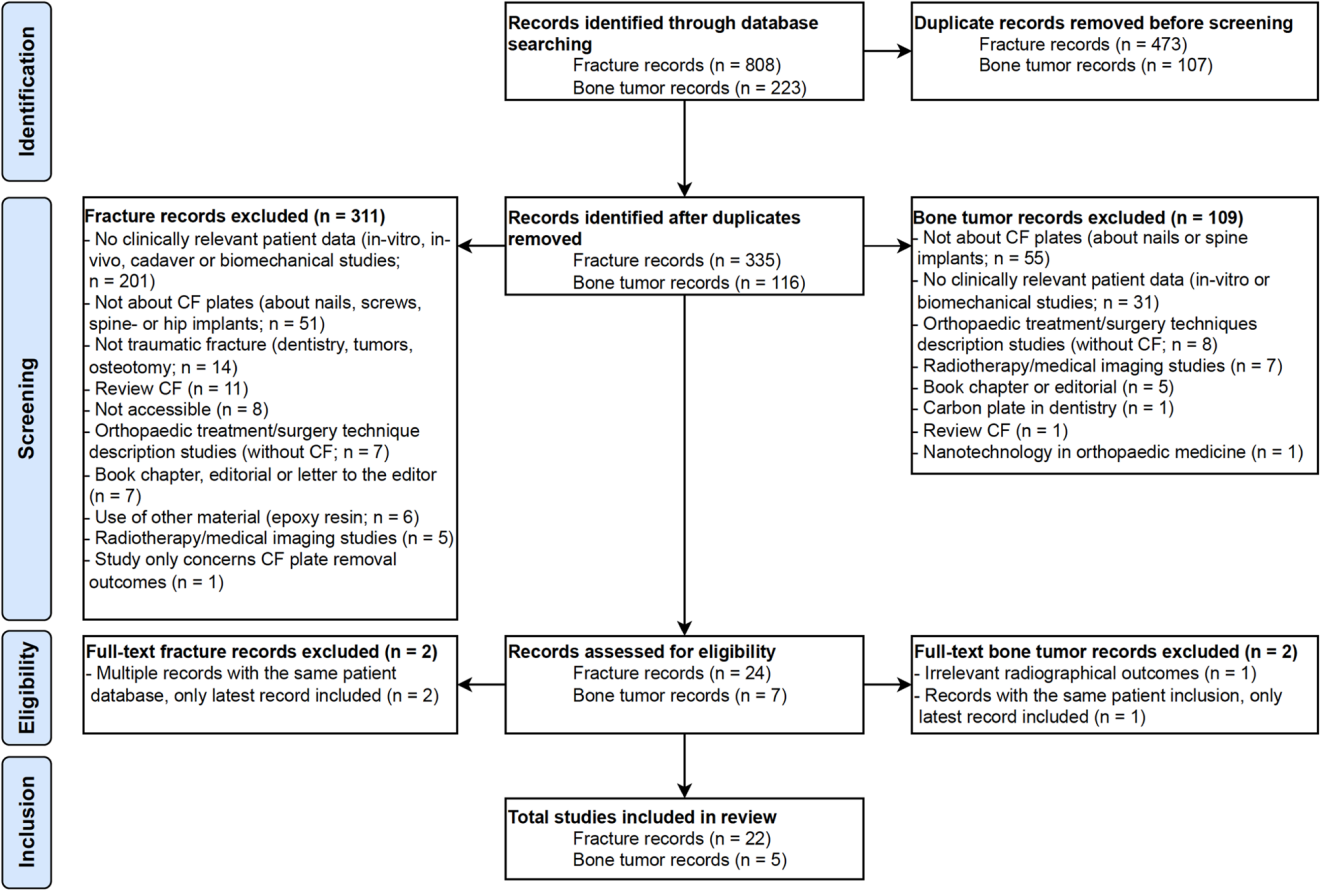
PRISMA flow diagram of the study. *CF* Carbon-fibre

After the retrieval of eligible studies, duplicates were removed. Out of the initial pool of 808 traumatic fracture records and 223 oncologic (bone tumour) records, a total of 335 studies on trauma fractures and 116 studies on (impending) pathological fractures remained. Abstracts were obtained and evaluated. Preliminary screening of titles and abstracts led to the exclusion of 311 studies for trauma fractures and 109 studies for (impending) pathological fractures. Subsequently, the full text of 24 studies on trauma fractures were reviewed, and 2 of them were excluded because a more recent third study used the same patient database. Similarly, two of the seven studies concerning (impending) pathological fractures were excluded after full-text screening: one due to irrelevant outcome measurements and one because the same patient had been included in a more recent study (Fig. 1).

### Quality assessment

Methodological quality assessment varied based on the study design. According to the Cochrane Handbook guidelines, the Risk of Bias II (RoB 2) tool was applied for RCTs, the Risk of Bias in Nonrandomized Studies of Intervention (ROBINS-I) tool for non-RCTs, and the Joanna Briggs Institute Checklist for case reports [19–21]. With the aid of these tools, various forms of bias were evaluated, including confounding bias, selection bias, bias in classification of intervention, bias due to deviations from intended interventions, bias due to missing data, bias in measurement of outcome and bias in selection of the reported results [19–21]. In addition, the Grading of Recommendations Assessment, Development and Evaluation (GRADE) approach was utilized to grade quality of evidence, which is important for assessing appropriateness and trustworthiness of recommendations done in the evaluated studies [22]. Within GRADE the following quality of evidence options are possible: high, moderate, low and very low. Randomized trials were initially rated high, observational studies low and other levels of evidence very low. However, high-quality evidence was downgraded if methodological flaws existed, and low-quality evidence could be upgraded when large effect sizes exist. Three reviewers (Z.R., A.W., S.D.) independently assessed the risk of bias for the included studies, discrepancies were discussed and the senior author (M.v.d.S.) was consulted in case of persistent disagreement.

### Data extraction

A standard data extraction form was used to collect relevant data from the included studies. The extraction form captured study characteristics (authors, year of publication, country, setting, title, number of included patients and level of evidence), patient characteristics [age, sex, smoking, body mass index (BMI), ASA classification, comorbidities, indication for CF plate fixation and number of patients that received a CF plate], and outcomes (complications, union, clinical, radiological and patient reported outcomes, as well as the duration of follow-up) [23].

### Data analysis

To summarize the findings in a quantitative form, complications were subdivided per anatomical region and presented separately for the upper and lower extremities, considering complications might depend on mechanical loading [24, 25]. Descriptive statistics were performed using SPSS v.24 (IBM Corp., Armonk, NY, USA). Demographics of all included studies were shown using medians for continuous variables, as demographic data contained outliers and skewed data due to the inclusion of case reports.

## Results

### Study characteristics

A total of 22 studies involving 800 patients with trauma fractures (median follow-up 12 months) and 5 studies involving 102 patients (median follow-up 12 months) with (impending) pathological fractures were included in the systematic review. Among them, 11 studies (497 patients, median follow-up 12 months), including three RCTs, compared CF plates with metal plates for trauma fractures (Table 1).

**Table 1:** Demographics of all included studies (*n* = 27) for trauma fractures (*n* = 22), of which 11 were comparative, and (impending) pathological fractures (*n* = 5)

Parameter	Median (range)/% (*n*)
Trauma fracture studies (*n* = 22; 800 patients)	
Number of patients	31 (1–160)
Patient age in years	58 (18–94)
Percentage of patient who were woman	66% (393)
BMI in kg/m^2^*	28 (16–44)
Follow-up in months*	12 (1–48)
(Impending) pathological fracture studies (*n* = 5; 102 patients)	
Number of patients	2 (1–96)
Patient age in years	30 (2–77)
Percentage of patient who were woman	61% (62)
BMI in kg/m^2^*	24 (20–27)
Follow-up in months*	12 (6–35)
Comparative studies (*n* = 11; 497 patients)	
Number of patients	42 (22–87)
Patient age in years	59 (18–89)
Percentage of patient who were woman	64% (317)
BMI in kg/m^2^*	27 (19–48)
Follow-up in months*	16 (2–36)

*BMI*  body mass index; kg/m^2^  kilograms per square meter

^*^Not reported in all included studies

### Study quality

The overall quality assessment score for RCTs, according to RoB 2 tool was “some concerns” for all included RCTs (*n* = 3; Table 2). The ROBINS-I criteria score for non-comparative studies ranged from low to moderate (*n* = 19; Table 3), and the mean score for case reports was 6 out of 8 (*n* = 5; Table 4). Following the GRADE approach, randomized trials were initially rated with a high certainty of evidence. However, due to the risk of bias of the included RCTs, scores were lowered in their certainty of evidence to moderate (Table 5). Observational studies and case reports were rated as with a low or very low certainty of evidence (Table 5). Consequently, recommendations of using CF plates for fixating fractures should be done with caution.

**Table 2:** Risk of Bias II (RoB 2) tool for RCTs

Fracture studies	Randomization process	Deviations from intended interventions	Missing outcome data	Measurements of the outcome	Selection of the reported results	Overall bias judgement
Perugia [36]	Low	Low	Low	Low	Some concerns	Some concerns
Ziegler [29]	Some concerns	Low	Some concerns	Low	Some concerns	Some concerns
Berger-Groch [37]	Low	Low	Low	Low	Some concerns	Some concerns

Scoring: low risk, some concerns or high risk

**Table 3:** Risk of Bias in Nonrandomized Studies of Intervention (ROBINS-I) tool for non-RCTs

Study	Confounding	Selection of participants	Classification of interventions	Deviation from intended interventions	Missing data	Measurements of outcomes	Selection of reported results	Overall
Baker et al. [39]	NI	Low	Moderate	Low	Low	Moderate	Low	Moderate
Rotini et al. [16]	NI	Low	Moderate	Low	Moderate	Moderate	Low	Moderate
Maggio et al. [34]	NI	Low	Moderate	Low	Moderate	Moderate	Low	Moderate
Pinter et al. [45]	Moderate	Low	Moderate	Low	Moderate	Moderate	Low	Moderate
Allemann et al. [32]	NI	Moderate	Moderate	Low	Low	Moderate	Low	Moderate
Tarallo et al. [10]	NI	Low	Moderate	Low	Moderate	Moderate	Low	Moderate
Guzzini et al. [38]	NI	Moderate	Moderate	Low	Moderate	Moderate	Low	Moderate
Paracuollo et al. [35]	NI	Low	Moderate	Low	Low	Moderate	Low	Moderate
Caforio et al. [43]	NI	Moderate	Low	Low	Low	Moderate	Moderate	Moderate
Rijs et al. [47]	Moderate	Moderate	Moderate	Low	Low	Moderate	Low	Moderate
Schliemann et al. [28]	NI	Moderate	Moderate	Low	Low	Moderate	Low	Moderate
Guzzini et al. [44]	NI	Low	Low	Low	Moderate	Low	Low	Moderate
Katthagen et al. [26]	NI	Moderate	Low	Low	Low	Low	Low	Moderate
Mitchell et al. [42]	Moderate	Moderate	Moderate	Low	Low	Low	Low	Moderate
Padolino et al. [27]	Moderate	Moderate	Moderate	Low	Low	Low	Low	Moderate
Byun et al. [40]	Moderate	Moderate	Moderate	Low	Low	Low	Low	Moderate
Hazra et al. [30]	Moderate	Moderate	Moderate	Low	Low	Low	Low	Moderate
Behrendt et al. [33]	Moderate	Moderate	Low	Low	Low	Low	Low	Moderate
Kimmeyer et al. [31]	Moderate	Low	Moderate	Low	Moderate	Moderate	Low	Moderate

**Table 4:** Joanna Briggs Institute Critical Appraisal Checklist for Case Reports (*n* = 5)

JBI checklist questions	Fracture	Tumour			
	Mellon [41]	Laux [46]	Barnds [48]	Zoccali [50]	Yeung [49]
1. Were the patient’s demographic characteristics clearly described?	Yes	Yes	Yes	Yes	Yes
2. Was the patient’s history clearly described and presented as a timeline?	No	No	Yes	No	Yes
3. Was the current clinical condition of the patient on presentation clearly described?	Yes	Yes	Yes	Yes	Yes
4. Were diagnostic tests or assessment methods and the results clearly described?	Yes	Yes	Yes	Yes	Yes
5. Was the intervention(s) or treatment procedure(s) clearly described?	No	No	Yes	Yes	No
6. Was the post-intervention clinical condition clearly described?	Yes	Yes	Yes	Yes	Yes
7. Were adverse events (harms) or unanticipated events identified and described?	Yes	Yes	Yes	Yes	Yes
8. Does the case report provide takeaway lessons?	Yes	Yes	No	No	No
Overall appraisal	Included	Included	Included	Included	Included

Scoring: yes, no, unclear or not applicable. *JBI* Joanna Briggs Institute

**Table 5:** Reported complications

Trauma fracture fixation										
Study	Study design	Level of evidence	Quality of evidence (GRADE)	Number of patients*	Age**	Gender	Anatomic region of the plate	Indication	Follow-up (in months)	Complications
Dey Hazra [30]	RCS	III	Moderate	65 (30 CF)	61	22/30 female	Proximal humerus	Proximal humeral fracture	32	Structural complication [CF (*n* = 1) versus titanium (*n* = 1)]; Soft tissue complication [CF (*n* = 2) versus titanium (*n* = 0)]; humeral head necrosis [CF (*n* = 0) versus titanium (*n* = 3)]
Katthagen [26]	PCS	III	Low	42 (21 CF)	67	14 out of 21 female	Proximal humerus	Proximal humeral fracture	12	Soft tissue complications [CF (*n* = 4) versus titanium *n* = 0)]
Kimmeyer [31]	RCS	III	Low	98	66	74 out of 98 female	Proximal humerus	Proximal humeral fracture	28	Avascular necrosis (*n* = 12); head shaft malreduction (*n* = 12); soft tissue complications (*n* = 7); structural complications (*n* = 5); tuberosity malreduction (*n* = 5); malreduction of the fracture (*n* = 3); tuberosity resorption/dislocation (*n* = 2); secondary glenohumeral osteoarthritis (*n* = 2); infection (*n* = 1)
Padolino [27]	RCS	III	Low	42 (21 CF)	57	12 out of 21 female	Proximal humerus	Proximal humeral fracture	31	Structural complication [CF (*n* = 2) versus titanium (*n* = 0)]; humeral head necrosis [CF (*n* = 1) versus titanium (*n* = 1)]; tuberosity resorption [> 50%; CF (*n* = 3) versus titanium (*n* = 9)]; varus/valgus malalignment [CF (*n* = 2) versus titanium (*n* = 0)]
Rotini [16]	PCaS	III	Low	160	64	s1 19 out of 160 female	Proximal humerus	Proximal humeral fracture	24	Structural complication (*n* = 15); soft tissue complication (*n* = 39); humeral head necrosis (*n* = 13); reduction loss/tuberosity dislocation (*n* = 7); nonunion (*n* = 2)
Schliemann [28]	RCS	III	Low	58 (29 CF)	66	22 out of 29 female	Proximal humerus	Proximal humeral fracture	24	Humeral head necrosis [CF (*n* = 1) versus metal (*n* = 3)]; varus malalignment [CF (*n* = 4) versus metal (*n* = 7)]
Ziegler [29]	RCT	II	Moderate	63 (32 CF)	62	26 out of 32 female	Proximal humerus	Proximal humeral fracture	6	None
Allemann [32]	RCaS	IV	Low	10	53	4 out of 10 female	Distal radius	Distal radius fracture	12	None
Behrendt [33]	PCS	III	Low	26 (14 CF)	57	11 out of 14 female	Distal radius	Distal radius fracture	2	None
Berger-Groch [37]	RCT	II	Moderate	31 (16 CF)	59	10 out of 16 female	Distal radius	Distal radius fracture	36	Soft tissue complications [CF (*n* = 1) versus titanium (*n* = 2)]
Di Maggio [34]	RCaS	IV	Low	64	57	38 out of 64 female	Distal radius	Distal radius fracture	12	None
Paracuollo [35]	RCaS	IV	Low	40	62	22 out of 40 female	Distal radius	Distal radius fracture	12	None
Perugia [36]	RCT	II	Moderate	30 (15 CF)	57	10 out of 15 female	Distal radius	Distal radius fracture	16	None
Tarallo [10]	RCaS	IV	Low	110	58	77 out of 110 female	Distal radius	Distal radius fracture	48	Structural complication (*n* = 5); soft tissue complication (*n* = 3); infection (*n* = 1)
Guzzini [38]	PCaS	III	Low	22	51	14 out of 22 female	Distal radius	Distal radius fracture	12	Soft tissue complication (*n* = 1)
Baker [39]	RCaS	IV	Low	12	78	*Not reported*	Proximal femur	THA periprosthetic fracture	*Not reported*	Nonunion (*n* = 1)
Byun [40]	RCS	III	Low	31 (10 CF)	50	3 out of 10 female	Distal femur	Distal femur fracture	6	None
Mellon [41]	CR	IV	Very low	1	64	1 out of 1 female	Distal femur	Distal femur fracture	1	Structural complication (*n* = 1)
Mitchell [42]	RCS	III	Low	22 (11 CF)	72	8 out of 11 female	Distal femur	Distal femur fracture	12	Structural complications [CF (*n* = 0) versus stainless steel (*n* = 4)]; nonunion [CF (*n* = 1) versus stainless steel (*n* = 4)]
Caforio [43]	PCaS	IV	Low	27	57	13 out of 27 female	Distal fibula + distal tibia	Ankle fracture	4	Soft tissue complication (*n* = 1)
Guzzini [44]	PCS	III	Low	87 (47 CF)	57	32 out of 46 female	Distal fibula + distal tibia	Ankle fracture	24	Infection [CF (*n* = 3) versus stainless steel (*n* = 4)]
Pinter [45]	RCaS	IV	Low	30	47	18 out of 30 female	Distal fibula	Unstable lateral malleolus fracture	20	Soft tissue complication (*n* = 1); infection (*n* = 1); nonunion (*n* = 1)
(Impending) pathological fracture fixation										
Laux [46]	CR	IV	Very low	2	77	2 out of 2 male	Humerus and tibia	Pathological fracture and prophylactic plate after curettage	6 and 8	None
Zoccali [50]	CR	IV	Very low	1	3	1 out of 1 female	Femur	Plate fixation after reconstruction	12	None
Yeung [49]	CR	IV	Very low	2	60	2 out of 2 female	Femur	Plate fixation after reconstruction	12 and 15	None
Rijs [47]	RCaS	IV	Low	96	43	59 out of 96 female	Femur (*n* = 67), tibia (*n* = 14), humerus (*n* = 13) and radius (*n* = 2)	(Impending) pathological fractures and plate fixation after reconstructions	35	Structural complication (*n* = 7); infection (*n* = 4); soft tissue complication (*n* = 1); tumour progression (*n* = 5); aseptic loosening (*n* = 1); nonunion (*n* = 2); angular deformation (*n* = 2)
Barnds [48]	CR	IV	Very low	1	9	1 out of 1 male	Tibia	Plate fixation after reconstruction	3	Structural complication (*n* = 1)

*THA* total hip arthroplasty; CF = carbon-fibre; RCS = retrospective cohort study; *RCaS* = retrospective case study; *PCS*  prospective cohort study, *PCaS*  prospective case study, *CR*  case report(s), *RCT*  randomized controlled trial

^*^Number of patients treated with carbon-fibre plates between brackets

**Mean or median age (as reported in the study)

### Reported complications after CF plate fixation for trauma fractures

In the upper extremity, seven studies evaluated CF plate fixation after traumatic proximal humerus fractures, involving a total of 391 patients [16, 26–31]. The most frequently reported complications were soft tissue complications (*n* = 52; 13%), including impingement between plate and acromion (*n* = 18), rotator cuff lesions (*n* = 18), adhesive capsulitis/shoulder stiffness (*n* = 15) and an intra-articular bicep tendon rupture (*n* = 1). Avascular humeral head necrosis/collapse was also frequently reported (*n* = 27; 7%). In addition, structural complications were frequently observed (*n* = 23; 6%), which consisted of secondary screw perforation (*n* = 12), screws backing out (*n* = 5), plate breakages (*n* = 4) and malpositioning of the plate (*n* = 2). Furthermore, secondary loss of reduction or resorption (> 50%) of tuberosity (*n* = 17; 4%), varus/valgus malalignment (*n* = 6; 2%), head shaft malreduction (*n* = 12; 3%), malreduction of the fracture (*n* = 3; 1%), nonunions (*n* = 2; 1%), secondary glenohumeral osteoarthritis (*n* = 2; 1%) and an infection (*n* = 1; < 1%) were documented as unfavourable events. Eight studies reported on traumatic distal radius fractures, with a total of 291 patients [10, 32–38]. Complications for this group included structural complications (*n* = 6; 2%), soft tissue complications (*n* = 5; 2%) and an infection (*n* = 1; < 1%).

Regarding the lower extremity, four studies assessed traumatic femur fracture fixations with CF plates, encompassing a total of 34 patients [39–42]. Complications observed in this group included one nonunion (*n* = 1; 3%) and one structural complication (plate breakage, *n* = 1; 3%). Furthermore, three studies evaluated ankle fractures treated with CF plates [43–45], involving 104 patients in total. The most frequently reported complications included infections (*n* = 4; 4%), soft tissue complication (*n* = 2; 2%) and one nonunion (*n* = 1; 1%; Table 5).

### Reported complications after CF plate fixation for (impending) pathological fractures

In the upper extremity, two studies evaluated pathological fractures involving 14 humerus and 2 distal radius CF plates [46, 47]. Most frequently reported humerus complications included infections (*n* = 2; 14%), a structural complication (traumatic plate breakage, *n* = 1; 7%) and a tumour progression (*n* = 1; 7%) for which the plate was removed. No complications were reported for the 2 distal radius CF plates.

Regarding the lower extremity, five studies encompassing a total of 86 patients investigated femoral and/or tibial (impending) pathological fractures [46–50]. Complications included structural failures (*n* = 6; 7%), consisting of plate breakages without clear trauma (*n* = 2), periprosthetic fractures (*n* = 2), screw breakage (*n* = 1) and screw backing out (*n* = 1). Additionally, documented complications consisted of tumour progressions (*n* = 5; 6%), infections (*n* = 4; 5%), nonunion (*n* = 3; 4%), aseptic loosening (*n* = 2; 3%), paediatric complications (valgus deformations treated with eight-plates, *n* = 2; 3%) and a soft tissue complication (wound dehiscence after radiotherapy treatment, *n* = 1; 2%; Table 5).

### Studies comparing CF plates with metal plates

Eleven studies have compared CF plates with metal plates, all focusing on traumatic fractures [26–30, 33, 36, 37, 40, 42, 44]. Among these studies, three were RCTs, and the remaining eight were prospective (*n* = 4) or retrospective (*n* = 4) comparative studies. This study hypothesized that CF plates display similar complication rates and clinical outcomes as metal plates for fracture fixation.

In the upper extremity, five studies examined CF plates compared with metal plates for humerus fractures. Firstly, Dey Hazra et al. conducted a retrospective study comparing range of motion after 2 years after fixation using CF plates (*n* = 30) or titanium plates (*n* = 35) [30]. The CF group demonstrated significantly improved forward flexion, internal rotation and abduction compared with the titanium group, with similar patient reported outcomes. Secondly, Katthagen et al. prospectively enrolled 21 CF-treated patients and matched them with 21 titanium treated patients [26]. Although functional outcomes were comparable after 12 months, the titanium group required more revisions due to screw perforations (5 versus 0; *p* = 0.048). Thirdly, Schliemann et al. conducted a prospective study comparing clinical and radiographic results of CF-treated patients (*n* = 29) to those treated with metal locking plates (*n* = 29) [28]. After 2 years, patients treated with CF plates achieved significantly better Constant Murley and Oxford Shoulder scores (*p* = 0.038 and 0.029, respectively), with fewer cases with loss of reduction or varus deformity in the CF group. Fourthly, Padolino et al. conducted a retrospective study comparing clinical and radiographic outcomes of CF-treated patients (*n* = 21) to those treated with titanium plates (*n* = 21) [27]. Shoulder mobility, clinical and pain scores were similar in both patient groups after 2 years, while cortical thinning was significantly greater in the CF group (*p* = 0.0003). Besides, the metal group exhibited a significantly higher rate of tuberosity resorption (*p* = 0.040). Lastly, Ziegler et al. performed an RCT comparing CF plates (*n* = 32) with titanium plates (*n* = 31), but reported no clinical or radiological differences after 6 month’s follow-up [29]. For distal radius fractures, three comparative studies consistently demonstrated similar clinical and radiological outcomes during follow-up evaluations spanning 2 weeks to 3 years [33, 36, 37].

In the lower extremity, two studies evaluated CF and metal plates for distal femur fractures. Mitchell et al. compared CF plates (*n* = 11) with stainless steel plates (*n* = 11), observing a trend towards better outcomes in the CF plate group, including less nonunion, less structural failures and less reoperations (9% versus 36%; 0% versus 18%; and 9% versus 36%, respectively) [42]. Byun et al. also compared CF (*n* = 10) with stainless steel (*n* = 21), noting better callus formation at 3 months, although this effect diminished at 6 months [40]. Regarding ankle fractures, Guzzini et al. compared CF plates (*n* = 47) with stainless steel plates (*n* = 41), reporting no significant differences in terms of pain, radiographic and clinical outcomes at 6-, 12- and 24-month follow-up evaluations [44] (Table 5).

## Discussion

As hypothesized, the findings of this systematic review indicate that utilization of CF plates for the fixation of traumatic, and (impending) pathological fractures is associated with a comparable incidence of complications and clinical outcomes to conventional metal plates. CF implants have gained increasing interest due to their potential advantages over metal implants. These advantages include radiolucency, which allows for improved visualization of bone healing and early detection of tumor recurrence, ensuring timely interventions if necessary. The absence of metallic artefacts on radiographic imaging enables more precise postoperative radiotherapy planning. Other advantages include reduced stress shielding which potentially leads to better bone quality, and the absence of cold welding, which facilitates easier removal [1–6, 8, 10]. The reported complication data can serve as a valuable benchmark for clinicians and patients, helping manage expectations during CF plate treatment. Although existing evidence suggests CF plates are a viable addition to the surgeons’ armamentarium, quality of current evidence is moderate to weak. Hence, recommendations of utilizing CF plates instead of conventional metal plates should be done with caution.

Adoption of CF plates as standard care for fracture fixation may face challenges due to the well-established use of conventional metal plates and the surgeons’ extensive training and experience with these conventional plates [51]. New technologies are often associated with a learning curve, as performance tends to improve over time [52, 53]. Nevertheless, the surgical procedure in terms of operation time and accuracy of implant position was similar in CF plates compared with metal plates [31, 33]. Moreover, comparable rates of reported complications suggest that implementation of CF plates does not necessitate additional training. Costs of innovations are another important factor for implementation. Although there is a lack of cost-effectiveness studies for CF plates, a recent study comparing CF nails with metal nails showed comparable cost profiles [54]. Yet, long-term evidence on safety and effectiveness needs to be further investigated before adaptation on a large scale is feasible. Rotini et al. and Tarallo et al. both described intraoperative plate breakages at an oval screw hole in the first generation of CF plates [10, 16]. This issue was not reported in more recent studies. Still, one of the drawbacks of CF is the inability to bend the plate to match the patient’s surface anatomy during surgery. Therefore, good preoperative planning is recommended when using these implants. Importantly, patients should be involved in the decision-making and evaluation of implant material, and other osteosynthesis methods, such as intramedullary nailing, should be considered before definitive treatment [55, 56].

Three systematic reviews have previously evaluated CF plates for trauma fracture fixation. Firstly, Saracco et al. included seven studies on distal radius fractures, and reported CF as a potential alternative to conventional metal plates [12]. Secondly, Theivendran et al. evaluated CF fixation in a broader population with small improvements in functional recovery of CF plates after humerus fractures, while there was insufficient evidence to support its widespread use [13]. Thirdly, Choloros et al. (9 studies, 361 patients) states that, considering their high union rates in extremity fracture fixation, CF seems to be a valid alternative to conventional metal plating [11]. Our systematic review (27 studies, 1297 patients), which also included pathological fractures, aligns with these previous results, and reported comparable material specific complications to their metal counterparts. However, high-quality RCTs with long-term follow-up are strongly recommended to provide additional evidence supporting the use of CF plates, their hypothesized advantages and possible contraindications.

### Limitations

This systematic review has several limitations. First, its quality is inherently related to the quality of the included studies. Level I or II comparative studies were limited, which represents a major limitation. In general, Level III and IV studies are more prone to selection bias (related to patient selection and/or uncontrolled confounders). The moderate to weak outcomes of the risk of bias assessment and GRADE approach to rate quality of evidence reflected our methodological concerns. However, all studies were still included because we wanted to provide a thorough overview of all available literature. Second, the lack of high-quality studies comparing CF and metal plates was a notable limitation. Especially for (impending) pathological fractures, the absence of comparative studies is a drawback which invites future research. Third, due to the lack of homogenous (comparative) studies and heterogeneity in patient populations, cancer types and complications, a meta-analysis was not performed. Pooling results with data on different complications and types of trauma or cancers would yield results with limited clinical validity. Fourth, there was a lack of clarity between minor and major (complications requiring surgical) interventions, which also limited our reporting about complications. Lastly, most of the included studies only reported short- or midterm follow-up results, which hampers our ability to draw conclusions on the long-term safety and effectiveness of CF plates. Further research is needed to generate high-quality evidence on the long-term safety and effectiveness of CF plates compared with metal plates. Nevertheless, this review provides a comprehensive overview with a complete up-to-date summary on the complication profile of CF plates in traumatic and (impending) pathological fractures.

## Conclusion

This systematic review hypothesized that CF plates display similar complication rates and clinical outcomes as metal plates for fracture fixation. Based on the available evidence, this systematic review concludes that CF plates are a viable alternative to metal plates for fracture fixation, without increased material-specific complications. However, more high-quality studies are needed to strengthen the evidence, especially for (impending) pathological fractures. In the meantime, the study’s complication data can serve as a valuable benchmark for clinicians and patients, helping manage expectations during CF plate treatment.

## Data Availability

All data used for analysis in this study was public.

## Appendix 1

Search strategy PubMed, search strategies other databases and their results are available upon request.

### Fracture; 132 results in PubMed from database inception up until 20 June 2023.

((“carbon fiber reinforced polyetheretherketon plate”[tw] OR “carbon fiber reinforced polyetheretherketon plates”[tw] OR “carbon fiber reinforced polyetheretherketon”[tw] OR “carbon fiber reinforced polyether ether ketone”[tw] OR “carbon fiber reinforced poly ether ether ketone”[tw] OR “Carbon fiber reinforced poly etheretherketone”[tw] OR “CFR PEEK plates”[tw] OR “CFR PEEK plate”[tw] OR “CFR PEEK”[tw] OR “CFR PEEK*”[tw] OR “CFRPEEK”[tw] OR “CFRPEEK*”[tw] OR “Carbon Fiber Reinforced PEEK”[tw] OR “carbon peek”[tw] OR “Carbon fiber plates”[tw] OR “Carbon fiber plate”[tw] OR “CF plates”[tw] OR “CF plate”[tw] OR “Carbon fiber implants””[tw] OR “Carbon fiber implant”[tw] OR “CF implants”[tw] OR “CF implant”[tw] OR ((“carbon fiber*”[tw] OR “carbonfiber*”[tw] OR “CFR”[tw]) AND (“polyetheretherketon*”[tw] OR “polyether ether keton*”[tw] OR “poly ether ether ketone”[tw] OR “poly etheretherketone”[tw] OR “PEEK”[tw])) OR ((“Carbon Fiber”[Mesh] OR “Carbon”[Mesh] OR “carbon fiber”[tw] OR “carbon fibers”[tw] OR “carbon fibre”[tw] OR “carbon fibres”[tw]) AND (“Bone Plates”[Mesh] OR “bone plate”[tw] OR “bone plates”[tw] OR “bone plating”[tw] OR “plate”[ti] OR “plates”[ti]))) AND (“Fractures, Bone”[Mesh] OR “Fractures”[tw] OR “Fracture”[tw] OR “Fractur*”[tw] OR “break”[tw] OR “breaks”[tw] OR “broken”[tw] OR “broke”[tw] OR “malunion*”[tw] OR “mal union*”[tw] OR “nonunion*”[tw] OR “non union*”[tw]) NOT (“Animals”[mesh] NOT “Humans”[mesh]) AND english[la]).

### Bone tumor; 44 results in PubMed from database inception up until 20 June 2023.

((“carbon fiber reinforced polyetheretherketon plate”[tw] OR “carbon fiber reinforced polyetheretherketon plates”[tw] OR “carbon fiber reinforced polyetheretherketon”[tw] OR “carbon fiber reinforced polyether ether ketone”[tw] OR “carbon fiber reinforced poly ether ether ketone”[tw] OR “Carbon fiber reinforced poly etheretherketone”[tw] OR “CFR PEEK plates”[tw] OR “CFR PEEK plate”[tw] OR “CFR PEEK”[tw] OR “CFR PEEK*”[tw] OR “CFRPEEK”[tw] OR “CFRPEEK*”[tw] OR “Carbon Fiber Reinforced PEEK”[tw] OR “carbon peek”[tw] OR “Carbon fiber plates”[tw] OR “Carbon fiber plate”[tw] OR “CF plates”[tw] OR “CF plate”[tw] OR “Carbon fiber implants”[tw] OR “Carbon fiber implant”[tw] OR “CF implants”[tw] OR “CF implant”[tw] OR ((“carbon fiber*”[tw] OR “carbonfiber*”[tw] OR “CFR”[tw]) AND (“polyetheretherketon*”[tw] OR “polyether ether keton*”[tw] OR “poly ether ether ketone”[tw] OR “poly etheretherketone”[tw] OR “PEEK”[tw])) OR ((“Carbon Fiber”[Mesh] OR “Carbon”[Mesh] OR “carbon fiber”[tw] OR “carbon fibers”[tw] OR “carbon fibre”[tw] OR “carbon fibres”[tw]) AND (“Bone Plates”[Mesh] OR “bone plate”[tw] OR “bone plates”[tw] OR “bone plating”[tw] OR “plate”[ti] OR “plates”[ti]))) AND (“Bone Neoplasms”[Mesh] OR “Neoplasms, Bone Tissue”[Mesh] OR “Bone Neoplasm”[tw] OR “Bone Neoplasms”[tw] OR “Bone Malignancy”[tw] OR “Bone Malignancies”[tw] OR “Orthopaedic oncology”[tw] OR “Orthopedic oncology”[tw] OR “Orthopedic tumor”[tw] OR “Orthopedic tumors”[tw] OR “Orthopaedic tumor”[tw] OR “Orthopaedic tumors”[tw] OR “Orthopaedic tumour”[tw] OR “Orthopaedic tumours”[tw] OR “Bone tumor”[tw] OR “Bone tumors”[tw] OR “Bone tumour”[tw] OR “Bone tumours”[tw] OR “Bone cancer”[tw] OR “Bone cancers”[tw] OR “Adamantinoma”[tw] OR “Adamantinomas”[tw] OR “Osteochondroma”[tw] OR “Osteochondromas”[tw] OR “Giant cell tumor”[tw] OR “Giant cell tumors”[tw] OR “Giant cell tumour”[tw] OR “Giant cell tumours”[tw] OR “Osteoblastoma”[tw] OR “Osteoblastomas”[tw] OR “Ewing sarcoma”[tw] OR “Ewing sarcomas”[tw] OR “Ewings sarcomas”[tw] OR “Ewings sarcoma”[tw] OR “Ewing’s sarcomas”[tw] OR “Ewing’s sarcoma”[tw] OR “Soft tissue sarcoma”[tw] OR “Soft tissue sarcomas”[tw] OR “Osteosarcoma”[tw] OR “Osteosarcomas”[tw] OR “Femoral Neoplasm”[tw] OR “Femoral Neoplasms”[tw] OR “Femoral Tumor”[tw] OR “Femoral Tumors”[tw] OR “Femoral Tumour”[tw] OR “Femoral Tumours”[tw] OR “Jaw Cancer”[tw] OR “Jaw Malignancies”[tw] OR “Jaw Malignancy”[tw] OR “Jaw Neoplasm”[tw] OR “Jaw Neoplasms”[tw] OR “Jaw Tumor”[tw] OR “Jaw Tumors”[tw] OR “Jaw Tumour”[tw] OR “Jaw Tumours”[tw] OR “Mandibular Cancer”[tw] OR “Mandibular Malignancies”[tw] OR “Mandibular Malignancy”[tw] OR “Mandibular Neoplasm”[tw] OR “Mandibular Neoplasms”[tw] OR “Mandibular Tumor”[tw] OR “Mandibular Tumors”[tw] OR “Mandibular Tumour”[tw] OR “Mandibular Tumours”[tw] OR “Maxillary Cancer”[tw] OR “Maxillary Cancers”[tw] OR “Maxillary Malignancies”[tw] OR “Maxillary Malignancy”[tw] OR “Maxillary Neoplasm”[tw] OR “Maxillary Neoplasms”[tw] OR “Maxillary Tumor”[tw] OR “Maxillary Tumors”[tw] OR “Maxillary Tumour”[tw] OR “Maxillary Tumours”[tw] OR “Orbital Cancer”[tw] OR “Orbital Cancers”[tw] OR “Orbital Malignancies”[tw] OR “Orbital Malignancy”[tw] OR “Orbital Neoplasm”[tw] OR “Orbital Neoplasms”[tw] OR “Orbital Tumor”[tw] OR “Orbital Tumors”[tw] OR “Orbital Tumour”[tw] OR “Orbital Tumours”[tw] OR “Palatal Cancer”[tw] OR “Palatal Cancers”[tw] OR “Palatal Malignancies”[tw] OR “Palatal Neoplasm”[tw] OR “Palatal Neoplasms”[tw] OR “Palatal Tumor”[tw] OR “Palatal Tumors”[tw] OR “Palatal Tumour”[tw] OR “Palatal Tumours”[tw] OR “Skull Base Cancer”[tw] OR “Skull Base Cancers”[tw] OR “Skull Base Malignancies”[tw] OR “Skull Base Malignancy”[tw] OR “Skull Base Neoplasm”[tw] OR “Skull Base Neoplasms”[tw] OR “Skull Base Tumor”[tw] OR “Skull Base Tumors”[tw] OR “Skull Base Tumour”[tw] OR “Skull Base Tumours”[tw] OR “Skull Neoplasm”[tw] OR “Skull Neoplasms”[tw] OR “Skull Tumor”[tw] OR “Skull Tumors”[tw] OR “Skull Tumour”[tw] OR “Skull Tumours”[tw] OR “Spinal Cancer”[tw] OR “Spinal Malignancies”[tw] OR “Spinal Malignancy”[tw] OR “Spinal Neoplasm”[tw] OR “Spinal Neoplasms”[tw] OR “Spinal Tumor”[tw] OR “Spinal Tumors”[tw] OR “Spinal Tumour”[tw] OR “Spinal Tumours”[tw] OR “Spine Cancer”[tw] OR “Spine Cancers”[tw] OR “Spine Malignancy”[tw] OR “Spine Neoplasm”[tw] OR “Spine Neoplasms”[tw] OR “Spine Tumor”[tw] OR “Spine Tumors”[tw] OR “Spine Tumour”[tw] OR “Spine Tumours”[tw]) NOT (“Animals”[mesh] NOT “Humans”[mesh]) AND english[la]).

## Abbreviation

CF: Carbon-fibre
PRISMA: Preferred Reporting Items for Systematic Reviews and Meta-Analyses
BMI: Body mass index; measured in kg/m^2^; kilograms per square meter
ASA: American Society of Anesthesiologists Classification
RCT: Randomized controlled trial
ROB 2: Risk of bias II tool
ROBINS-I: Risk of Bias in Nonrandomized Studies of Intervention tool
GRADE: Grading of Recommendations Assessment, Development and Evaluation
